# Neuroprotective Effect for Cerebral Ischemia by Natural Products: A Review

**DOI:** 10.3389/fphar.2021.607412

**Published:** 2021-04-22

**Authors:** Qian Xie, Hongyan Li, Danni Lu, Jianmei Yuan, Rong Ma, Jinxiu Li, Mihong Ren, Yong Li, Hai Chen, Jian Wang, Daoyin Gong

**Affiliations:** ^1^ State Key Laboratory of Southwestern Chinese Medicine Resources, Chengdu, China; ^2^ School of Pharmacy, Chengdu University of Traditional Chinese Medicine, Chengdu, China; ^3^ Hospital of Chengdu University of Traditional Chinese Medicine, Chengdu, China

**Keywords:** natural products, cerebral ischemia, neuroprotection, therapeutic application, mechanisms

## Abstract

Natural products have a significant role in the prevention of disease and boosting of health in humans and animals. Stroke is a disease with high prevalence and incidence, the pathogenesis is a complex cascade reaction. In recent years, it’s reported that a vast number of natural products have demonstrated beneficial effects on stroke worldwide. Natural products have been discovered to modulate activities with multiple targets and signaling pathways to exert neuroprotection via direct or indirect effects on enzymes, such as kinases, regulatory receptors, and proteins. This review provides a comprehensive summary of the established pharmacological effects and multiple target mechanisms of natural products for cerebral ischemic injury *in vitro* and *in vivo* preclinical models, and their potential neuro-therapeutic applications. In addition, the biological activity of natural products is closely related to their structure, and the structure-activity relationship of most natural products in neuroprotection is lacking, which should be further explored in future. Overall, we stress on natural products for their role in neuroprotection, and this wide band of pharmacological or biological activities has made them suitable candidates for the treatment of stroke.

## Introduction

Stroke is a disease with high prevalence and incidence (Benjamin et al., 2018). Strokes can be divided into two categories: ischemic and hemorrhagic stroke, and over 80% are ischemic stroke (Zhou et al., 2018). Ischemic stroke is a pathological condition characterized by blood vessels occlusion and insufficient of blood supply. Stroke which is one of the most common causes of disability and death worldwide, seriously endangers human health and brings heavy burden to society and family (Donnan et al., 2008; Kalogeris et al., 2016). The pathological mechanism of cerebral ischemia is a complex cascade reaction, and its severity is related to the time of cerebral ischemia and the depth of the ischemic site (Steliga et al., 2020). The occurrence and development of cerebral ischemia included neuron excitotoxicity, mitochondrial dysfunction, neuroinflammatory damage, oxidative stress, etc. It is generally believed that glutamate excitotoxicity, energy metabolism disorder, and Ca^2+^ overload happened within 24 h of the onset of stroke, accompanied by the generation of free radicals (Guo, Li and Wang, 2009; Manzanero, Santro, Arumugam, 2013). Apoptosis and necrosis also occurred within a few hours of ischemia (Wang, C. et al., 2015). In the subacute phase, brain edema and blood-brain barrier (BBB) destruction happened. Endothelial cells, pericytes, astrocytes, etc are activated and inflammatory factors are released, accompanied with the proliferation of reactive glial cells (Stanimirovic and Satoh, 2000; Liebner et al., 2018). There exists endogenous repair in the late stage of cerebral ischemia, and neurogenesis, glial scars, angiogenesis could be observed (Steliga et al., 2020). Therefore, the occurrence and development of stroke is complicated, which makes its treatment very difficult. Tissue plasminogen activator (t-PA) is the only therapeutic medicine for ischemic stroke approved by Food and Drug Administration (FDA). However, t-PA has a restricted time window of 6 h, and delayed t-PA infusion increases the risk of hemorrhagic transformation and carries high mortality (Group 1995; Hacke et al., 2008). Despite constantly increasing understanding of ischemic stroke pathophysiology through laboratory and clinical studies, the treatment strategy is still not ideal. Therefore, there is an urgent medical need to identify new molecules that can provide neuroprotection against cerebral ischemic or ischemic reperfusion (I/R) injury.

**FIGURE 1:**
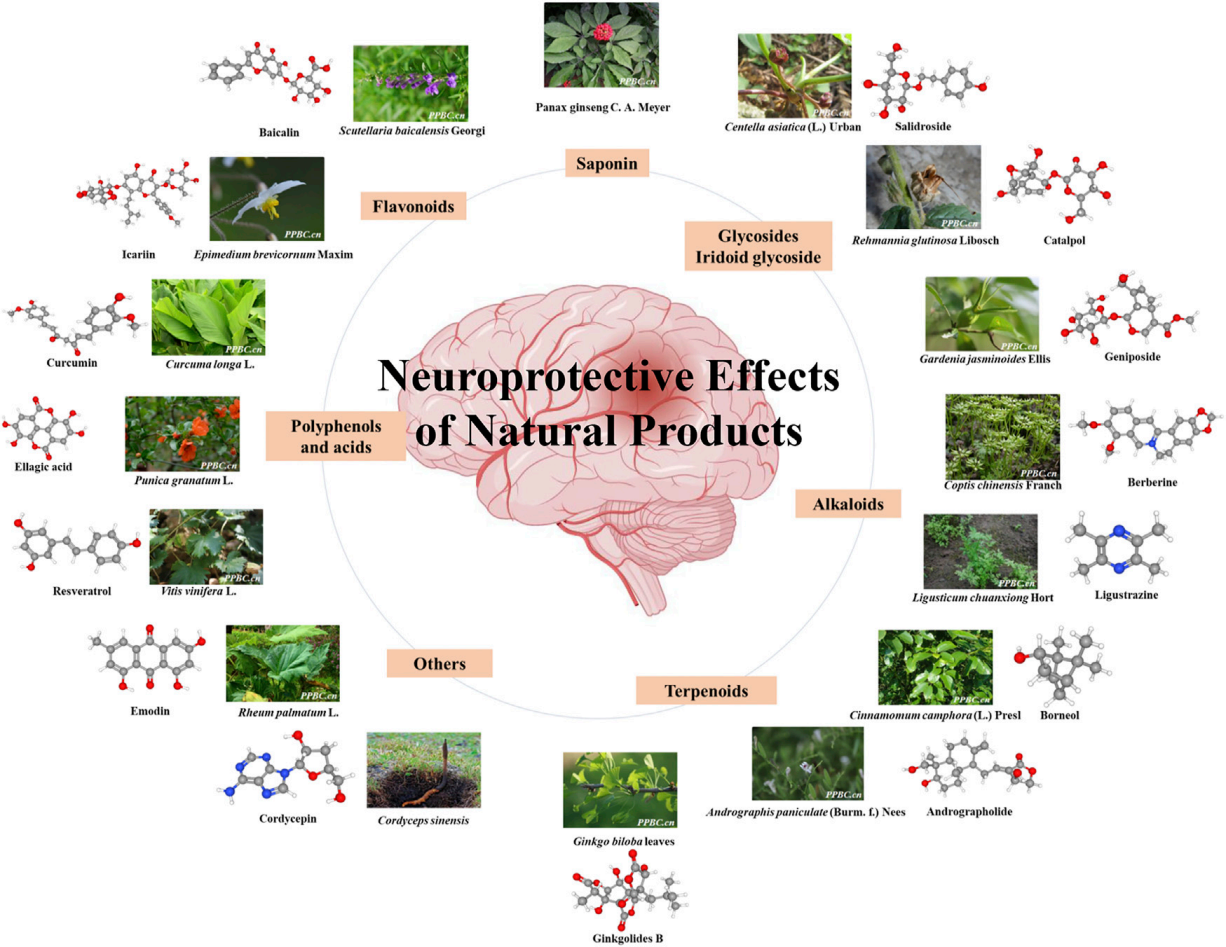
Neuroprotective effect of various natural products. The color figures of medicine are viewed in www.iplant.cn. The club model comes from PubChem.

Natural products obtained from natural sources, including plants, animals, fungi and microorganisms. Natural products have played an important role in ancient traditional medicine systems, such as Unani, Chinese and Ayurveda which are still in common use today. Natural products are known to exert additive, synergistic or antagonistic effects on the body. With the pharmacology technology developing, the administration forms of natural products are various, which has drawn much attention for the treatment of stroke from drug discovery to drug delivery (Hermann et al., 2019; Long et al., 2020; Williams et al., 2020). The pathogenesis of stroke is a complex cascade reaction, including neuro-inflammatory damage, oxidative stress, mitochondrial dysfunction, neurotoxicity, blood-brain barrier (BBB) disruption, neurovascular unit (NVU) damage and other factors. In recent years, it’s reported that a vast number of natural products have demonstrated beneficial effects on stroke worldwide (Fei et al., 2020). The neuroprotection of natural products has been discovered to modulate activities via multiple targets and signaling pathways with direct or indirect effects on enzymes, such as kinases, receptors and proteins (Bagli et al., 2016). This wide band of pharmacological or biological activities has made them suitable candidates for the treatment of stroke (Cai et al., 2019; Zhang, C. et al., 2020; Zhong et al., 2020). As a requirement of novel drug development for stroke, there is a focus on the neuroprotection of natural products via multi-targets. Hence, we stress on natural products for their role in neuroprotection (Figure 1). This review provides a comprehensive summary of the pharmacological effects and multiple target mechanisms of natural products for cerebral ischemic or I/R injury.

## Methodology

Database searches using Google scholar, PubMed, and China National Knowledge Internet (CNKI) were conducted until July 2020 to include up to date documented information in the present review. For data mining, the following words were used in the databases mentioned above: neuroprotection, natural product, phytoconstituents, natural products cerebral ischemic injury, natural products cerebral ischemic reperfusion injury, *in vivo* and *in vitro* studies for prevention and treatment of stroke. In almost all cases, the original articles or abstracts were obtained and the relevant data was extracted.

### Neuroprotective Role of Flavonoids in Ischemic Brain Injury

Flavonoids, a natural bioactive compound found abundant in vegetables, fruits and traditional herbal medicine. Prevention and/or treatment with flavonoids such as baicalin, apigenin, vitexin, quercetin and other flavonoid compounds have shown promising neuroprotective effects against ischemic-induced injury by through increasing neuronal viability, cerebral blood flow and reducing ischemic-related apoptosis (Putteeraj et al., 2018).

### Baicalin

**FIGURE 2:**
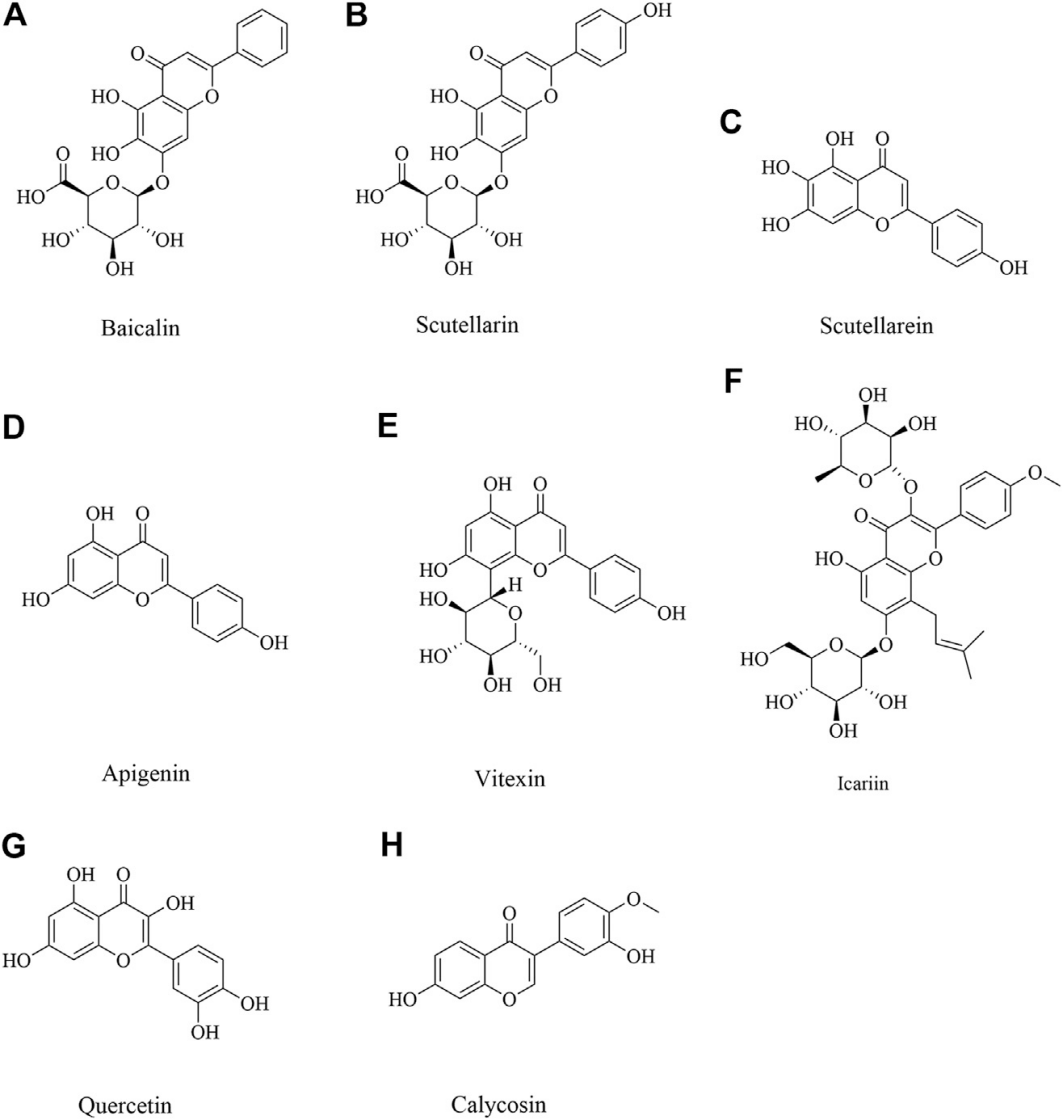
The flavonoids for cerebral ischemia injury.

Baicalin, mainly derived from the root of *Scutellaria baicalensis* Georgi, is one kind of a crucial flavonoid (Figure 2A). This medicinal plant is widely distributed in China, Russia, Mongolia, North Korea and Japan, and has the functions of clearing away heat and dampness, purging fire and detoxification (Zhao et al., 2019). In recent years, baicalin has also been found to have a wide range of neuroprotective effects (Liang et al., 2017; Sowndhararajan et al., 2018). Middle cerebral artery occlusion (MCAO) induced cerebral ischemic injury *in vivo* and oxygen–glucose deprivation (OGD) induced hypoxia injury *in vitro* were implemented. Baicalin at doses ranging from 50 to 200 mg/kg could significantly improve neurological deficit and reduce infarct volume via inhibiting apoptotic and oxidative pathway, including myeloid cell leukemia 1 (Mcl 1), B-cell lymphoma-2 (Bcl 2), superoxide dismutase (SOD), malondialdehyde (MDA), and so on (Cao et al., 2011; Zheng et al., 2015). Neuroprotective activity was also confirmed in another study. Baicalin could decrease nuclear factor kappa B (NF-κB) p 65 to exert neuroprotection (Xue et al., 2010). In addition, baicalin 100 mg/kg inhibits toll like receptor 2/4 (TLR2/4) signaling pathway in cerebral ischemia rats (Tu et al., 2011). Baicalin down-regulated nucleotide-binding oligomerization domain protein 2 (NOD2) receptor and tumor necrosis factor α (TNF α) expression of neurons with OGD *in vitro* and cerebral ischemia-reperfusion *in vivo* (Li, F et al., 2010). These findings suggested baicalin could afford neuroprotection through multiple pathways.

### Scutellarin and Scutellarein

Scutellarin (Figure 2B) and scutellarein (Figure 2C), is the major active component extracted from *Erigeron breviscapus* (Vant.) Hand-Mazz, a Chinese herbal medicine (Zhang et al., 2009). Accumulated data have demonstrated the effectiveness and benefits of breviscapine and scutellarin in treating cerebrovascular disease and cardiovascular disease in clinic and in experimental study (Cao et al., 2008; Wang et al., 2015a; Gao et al., 2017). It has demonstrated that scutellarin is benefit to brain injury caused by cerebral ischemia/reperfusion due to its anti-oxidation and anti-inflammatory effects and ability to attenuate neuronal damage (Wang and Ma 2018). Scutellarin could effectively suppress the inflammatory response in activated microglia/brain macrophage in experimentally induced cerebral ischemia, whose underlying mechanism was associated with suppressing the phosphory c-Jun N-terminal kinase (p-JNK) and p-p38 mitogen-activated protein kinase (MAPK), increasing phosphorylation extracellular regulated kinase 1/2 (p-ERK1/2) (Chen H.-L et al., 2020). In MCAO rats given scutellarin 100 mg/kg treatment, neurological scores were significantly improved coupled with a marked decrease in infarct size, which was found that scutellarin had a neuroprotective effect, amplified TNC1 astrogliosis and activated microglia to remodel injured tissue via notch pathway (Fang et al., 2015; Fang et al., 2016). Pretreated with scutellarin 50 or 75 mg/kg has protective effects for cerebral injury through upregulating endothelial nitric oxide synthase (eNOS) expression and downregulating of vascular endothelial growth factor (VEGF), basic fibroblast growth factor (bFGF), and inducible nitric oxide synthase (iNOS) expression (Hu et al., 2005). Another study also proved scutellarin protected against ischemic injury through regulation of nicotinamide adenine dinucleotide phosphate oxidase 2 (NOX2) and connexin 43 to decrease oxidative damage and apoptotic cell death (Sun, J. B. et al., 2018; Zhang et al., 2009). Scutellarin and scutellarein could improve neuronal injury, and scutellarein had better protective effect than scutellarin through improving the Ca^2+^-ATPase and Na^+^, K^+^-ATPase activity in rat cerebral ischemia with bilateral common carotid arteries (BCCAO) (Tang et al., 2014). Scutellarin protects brain from acute ischemic injury probably through its inhibitory effect on the angiotensin-converting enzyme (ACE) and Ang II type 1 receptor (AT1R), also TNF-α, interleukin-6 (IL-6), and interleukin-1β (IL-1β) (Wang et al., 2016).

### Apigenin and Vitexin

Apigenin (Figure 2D) is a natural flavonoid and vitexin (Figure 2E) is an apigenin flavone glycoside, which were found in several dietary plant foods as vegetables and fruits, and had a variety of pharmacological effects (Babaei et al., 2020). It has been reported that apigenin and vitexin played a protective role in diseases associated with the oxidative process, such as cardiovascular and neurological disorders (Ayoobi et al., 2017; Che et al., 2020; Li, S. et al., 2017). Angiogenesis is one of the ways to repair brain injury. It’s reported that apigenin could promote cell proliferation, tube formation, and cell migration while inhibiting apoptosis and autophagy by affecting caveolin-1/VEGF, Bcl-2, caspase-3, Beclin-1, and mechanistic target of rapamycin (mTOR) expression. What’s more, apigenin significantly reduced neurobehavioral scores and volume of cerebral infarction while promoting vascular endothelial cell proliferation by upregulating VEGFR2 to affect caveolin-1, VEGF, and eNOS expression in brain tissue of I/R rats (Pang et al., 2018). Vitexin could also play a protective role by suppressing oxidative stress (Cui et al., 2019). In addition, vitexin regulated cell apoptosis and autophagy via MAPK and mTOR/Ulk1 pathway to attenuate cerebral I/R injury (Wang et al., 2015b; Jiang et al., 2018). Apigenin also showed long-term therapeutic effect in cognitive impairments after cerebral I/R injury (Tu et al., 2017). Above all, it suggested apigenin and vitexin had neuroprotective effect through inflammation, angiogenesis, apoptosis and autophagy pathways with multi-targets.

### Icariin

Icariin (Figure 2F), an active flavonoid extracted from *Epimedium brevicornum* Maxim, has been proven to possess a wide range of efficacy including anti-tumor, anti-oxidant, anti-bacterial and anti-inflammatory, etc (Luo et al., 2007; Zhou et al., 2011). It has been also reported to alleviate brain injury and attenuate cognitive deficits (Li, H. et al., 2010; Li et al., 2005). Xiong et al. and Deng et al. found that pretreatment with Icariin and Icariside II, a metabolite of icariin, could decrease neurological deficit score, diminish the infarct volume, and reduce IL-1β and transforming growth factor-β (TGF-β1). Moreover, Icariin suppressed inhibitory κB (IκB)-α degradation and NF-κB activation and up-regulated peroxisome proliferator-activated receptor α (PPARα) and peroxisome proliferator activated receptor γ (PPARγ) levels in I/R model (Deng et al., 2016; Xiong et al., 2016). Moreover, a study by Mo et al. found that Icariin can inhibit apoptosis and inflammatory in neurons after OGD/R through inositol requiring enzyme 1 (IRE1)- X-Box Binding Protein 1 (XBP1) signaling pathway (Mo et al., 2020).

### Quercetin

Quercetin (Figure 2G), is a common flavonoid found in many fruits and vegetables (Lee et al., 2003). It has been reported that quercetin has an anti-oxidative property, anti - inflammation and immunity, anti-cancer, etc (Costa et al., 2016; Li, W. et al., 2016; Shafabakhsh and Asemi 2019; Xu, D. et al., 2019). A study by Jin et al. found that quercetin could increase the expression of ZO-1, Claudin-5, β-catenin, and LEF1, and decrease the expression of MMP-9, GSK-3β and axin to reduce brain edema and BBB leakage and improve BBB dysfunction, which suggested the neuroprotection of quercetin was related to Wnt/β-catenin pathway (Jin et al., 2019). Calcium acts as a second messenger that mediates physiologic functions. It’s reported that quercetin exerted a preventative effect through attenuation of intracellular calcium overload and restoration of down-regulated hippocalcin expression during ischemic injury (Park et al., 2020). Moreover, quercetin alleviated the increasement of protein tyrosine and serine/threonine phosphatase activity, along with the reduction of ERK and Akt phosphorylation in cerebral I/R injury (Wang, Y. Y. et al., 2020). In addition, iso-quercetin protected against oxidative stress and neuronal apoptosis via Nrf2-mediated inhibition of the NOX4/ROS/NF-κB signaling pathway in cerebral ischemic stroke (Dai et al., 2018).

### Calycosin

Calycosin (Figure 2H), an isoflavone phytoestrogen isolated from *Astragalus membranaceus*, is found to possess various potential pharmacological activities, such as, anti-tumorigenesis, anti-oxidation, anti-virus and apoptosis-modulation effects (Chen, J et al., 2015; Wang and Zhao, 2016; Zhao, Y. et al., 2016). It was also demonstrated that calycosin 30 mg/kg could ameliorate both the neurological deficit and infarct volume in experimental cerebral ischemia reperfusion injury, and the mechanism might be attributed to its antioxidant effects (Guo et al., 2012). Post-stroke calycosin 30 mg/kg therapy increased brain-derived neurotrophic factor (BDNF)/TrkB to ameliorate the neurological injury due to switching the microglia from the activated ameboid state to the resting ramified state in ischemic stroke rats (Hsu et al., 2020). Calycosin exhibited a downregulation of dexamethasone-induced Ras-related protein 1 (RASD1), and an upregulation of estrogen receptor (ER)-α and Bcl-2 (Wang, X. et al., 2014). A study by Wang et al. found that calycosin pretreatment for 14 days dramatically upregulated p62, neighbor of BRCA1 gene 1 (NBR1) and Bcl-2, and downregulated TNF-α to ameliorate neurological scores in cerebral I/R rats (Wang, L. et al., 2018).

### Neuroprotective Role of Alkaloids in Ischemic Brain Injury

Alkaloids are rated as among the most-investigated plant secondary metabolites with versatile biological activity (Qiu et al., 2014). Recent investigations have pointed out the dynamic role of alkaloids as antibacterial, antidepressant, antioxidant, anti-inflammation (Perviz et al., 2016; Li et al., 2020; Liu et al., 2020; Rosales et al., 2020). Accordingly, alkaloids are emerging as pharmaceutical tools for neuroprotective effects (An et al., 2020). Alkaloids were investigated on neuroprotective effects including berberine, ligustrazine, tetrahydropalmatine and others.

### Berberine

**FIGURE 3:**
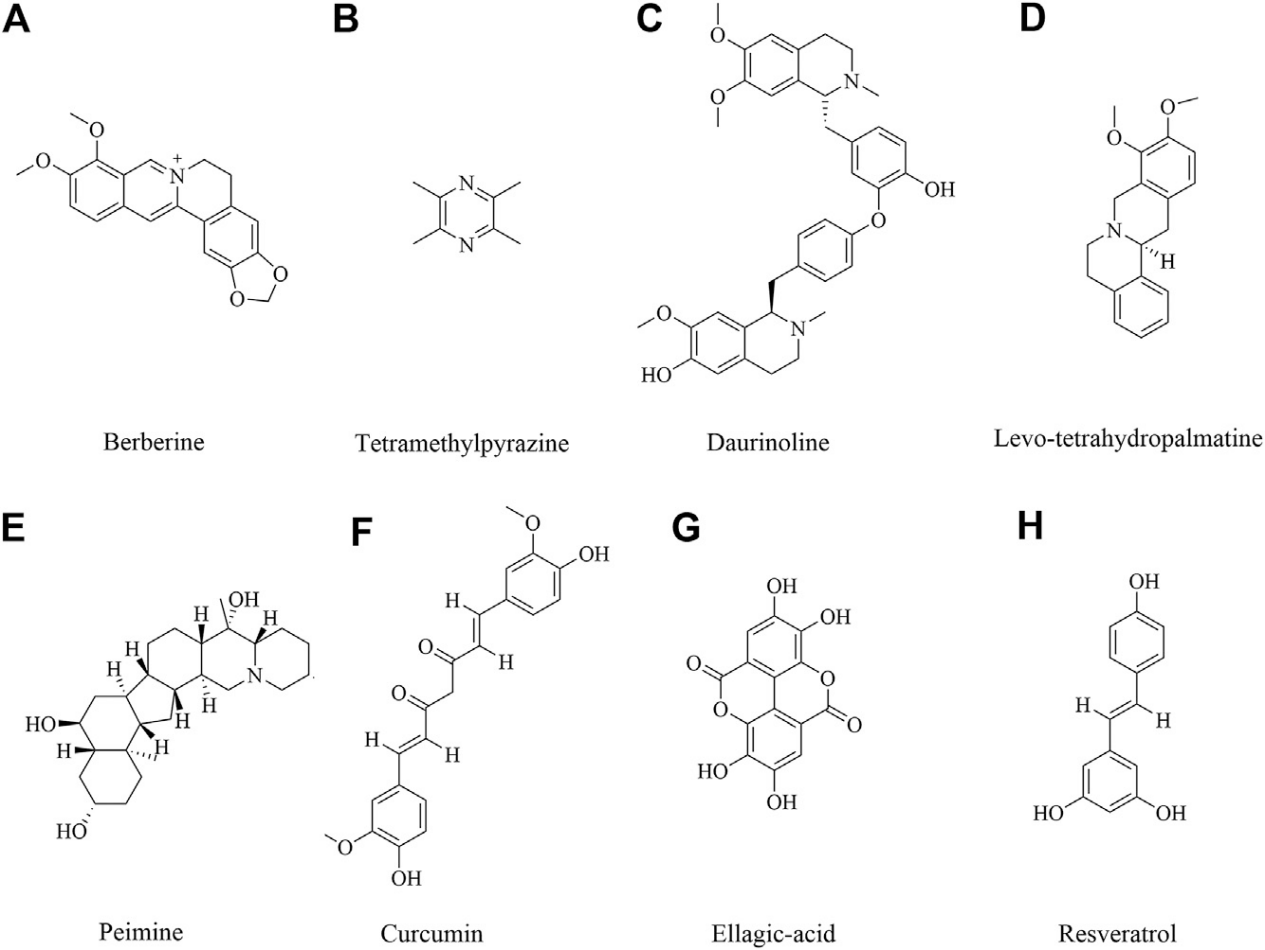
The alkaloids, polyphenols and acids for cerebral ischemia injury.

Berberine (Figure 3A) is an isoquinoline alkaloid isolated from traditional herbal medicine. It is widely present in the roots, rhizomes, stems or bark of various natural plants, such as *Coptis chinensis* Franch., *Phellodendron chinense* Schneid., etc. These plants have been used for thousands of years (Wang et al., 2019a; Fan et al., 2019). Studies have shown that berberine has a variety of pharmacological activities, including anti-microbial, anti-tumor, lowering glucose, lowering cholesterol, anti-inflammatory, antioxidant, immunomodulatory and neuroprotective effects (Asemi et al., 2020; Song et al., 2020). So far, the neuroprotective effect of berberine has been involved (Lin and Zhang 2018). Berberine could improve the survival rate in OGD/R cells and improve the neurological deficiency in cerebral ischemic injury, which was involved in reducing the expression of cleave caspase 3 and up-regulating the expression of p-Bad to inhibit cell apoptosis via TrkB-PI3K/Akt pathway to promote neuron growth (Hu et al., 2012; Chai et al., 2013; Yang et al., 2018a). Other studies also showed berberine 40 mg/kg preconditioning had neuroprotective effects by promoting autophagy, reducing cell apoptosis via PI3K/Akt signaling pathway (Zhang et al., 2016a; Zhang et al., 2016b). In addition, berberine exerted the neuroprotective effects by regulating I/R-induced peripheral lymphocytes early immunoactivation (Song et al., 2012). A study by Zhang et al. found that neuroprotection of early and short-time applying berberine could upregulate p-Akt, p-GSK 3β and phosphor cAMP response element binding protein (p-CREB) and downregulate NF-κB expression to ameliorate BBB permeability in the acute phase of cerebral ischemia (Zhang et al., 2012).

### Tetramethylpyrazine

Tetramethylpyrazine (Figure 3B), also called ligustrazine, is a kind of alkaloids identified in *Ligusticum chuanxiong* Hort, which has been used to treat cardiovascular and cerebrovascular diseases for hundreds of years in ancient China (Zhao, X. et al., 2016; Zheng, J. S. et al., 2018). In recent study, *Ligusticum chuanxiong* Hort and its main active compound ligustrazine have been studied and had anti-inflammatory, anti-oxidant and analgesic effect (Kao et al., 2006; Zou et al., 2018). Tetramethylpyrazine treatment with 20 mg/kg suppressed hypoxia inducible factor-1-alpha (HIF-1α), TNF-α, and activated caspase-3 expression in MCAO-induced brain ischemia in rats (Chang et al., 2007). Tetramethylpyrazine 20 mg/kg inhibited neutrophil activation via elevating nuclear related factor 2 (Nrf2) and heme oxygenase-1 (HO-1) expression and inhibiting high-mobility group box-1 protein (HMGB1)/toll-like receptor-4 (TLR4), Akt, and ERK pathway following cerebral ischemia rats (Chang et al., 2015). Tetramethylpyrazine analogues were also investigated to induce p-eNOS by activation of PI3K/Akt and TLR-4/NF-κB signaling pathway and regulate the expression of tight junction (TJ) proteins, matrix metalloprotein 9 (MMP-9) and aquaporin 4 (AQP4) under cerebral I/R injury (Yan et al., 2015; Xu et al., 2017; Zhou et al., 2019). The similar finds of PI3K/Akt pathway also were shown in another study (Ding et al., 2019). What’s more, the synergistic effect of drugs has also been shown to have good therapeutic effect. Ligustrazine accompanied with borneol could attenuate I/R injury by downregulating IL-1β, IL-6, and TNF-α, upregulating SOD, GSH-Px and regulating apoptosis and autophagy (Yu, B. et al., 2017; Yu et al., 2016).

### Daurinoline

Daurinoline (Figure 3C) is a kind of dibenzyl tetrahydroisoquinoline alkaloid, isolated from *menispermum dauricum* DC., which has a variety of pharmacological activities, including neuroprotective effect (Zhang et al., 2011). Daurinoline 10 mg/kg and 20 mg/kg could enhance of SOD activity and the ability of scavenging oxygen free radicals (Lan and Lianjun 2020). Daurinoline reduced the level of MDA in brain tissue and increase the activity of SOD to prevent and ameliorated energy metabolism disturbance of cortex and mitochondria of brain induced by repeatedly cerebral ischemia reperfusion (Li and Gong 2004). In addition, daurinoline 5 and 10 mg/kg could inhibit cytochrome c (Cyt-C) release and caspase-3 and caspase- 9 to inhibit neuronal cells apoptosis in the penumbra after cerebral ischemia (Yang et al., 2009).

### Levo-tetrahydropalmatine

Levo-tetrahydropalmatine (Figure 3D) are a series of alkaloids, isolated from a Chinese analgesic medicine, called *Corydalis yanhusuo* W. T. Wang. *l*-Tetrahydropalmatine, one of its main active ingredients, has been demonstrated to have potent analgesic effects and has been used in Chinese clinical practice for this purpose for many years (Chu et al., 2008; Han et al., 2012; Kang et al., 2016). Recent work showed that *l*-tetrahydropalmatine suppressed the central excitatory effects of amphetamine (Chang and Lin 2001), and played a protective role against neuronal injury in animal models (Liu et al., 2005; Mantsch et al., 2007; Chen et al., 2012). Levo-tetrahydropalmatine could regulate Src and c-Abl expression to inhibit neuron apoptosis and attenuate BBB injury in cerebral ischemic animals (Mao et al., 2015; Sun, J. B et al., 2018).

### Peimine

Peimine (Figure 3E) is a major biologically active component of *Fritillaria ussuriensis*. Recent studies have shown that isosteroidal alkaloids in Fritillaria had a wide range of pharmacological activities besides its role in cough remedies (Li, J. et al., 2019; Li et al., 2020a; Zhang et al., 2020a; Zhao et al., 2018). It’s also reported that peimine had protective effects on cerebral I/R injury in rats. The mechanism of neuroprotective effect might be related to inhibition of apoptosis, oxidative stress and inflammatory response, reduction of pathological damage and neurological dysfunction through regulation of the PI3K/Akt/mTOR pathway (Guo et al., 2019; Duan et al., 2020).

### Neuroprotective Role of Polyphenols and Acids in Ischemic Brain Injury

Polyphenols, a bioactive compound, were found abundant in vegetables, fruits and medical plant. Neuroprotection of polyphenols in medical plants is getting attention in the world. Curcumin, ellagic acid, resveratrol has been extensively studied and show multi-function. They are neuroprotectants, antioxidants, anti-inflammatory and antithrombic agents. It’s reported that the neuroprotective efficacy of these compounds was involved in mitigating brain infarction and global ischemia, improving cerebral blood circulation (Lin 2011).

### Curcumin

Curcumin (Figure 3F), a polyphenolic compound extracted from *Curcuma longa* L., has potential anti-inflammatory, cardiovascular protective effect and neuroprotection. Recent studies demonstrated the neuroprotective effect of curcumin against cerebral ischemic injury through oxidation, apoptosis, autophagy pathways (Eghbaliferiz et al., 2020; Forouzanfar et al., 2020; Ułamek-Kozioł et al., 2020). Rats or mice with cerebral ischemic injury that received curcumin at 10–400 mg/kg exhibited significantly alleviated brain injury. Moreover, a more SOD, GSH-Px and glutathione (GSH) and a lower MDA, NO contents were found in curcumin administrated animals (Thiyagarajan and Sharma 2004; Li. Y. et al., 2016), which suggested the mechanism of curcumin was related to antioxidative activity. In addition, curcumin was proven to suppress the release of inflammatory cytokines via NF-κB, signal transducer and activator of transcription (STAT), Akt/mTOR, ERK signaling pathways (Huang et al., 2018; Li et al., 2015; Mukherjee et al., 2019; Xu, H. et al., 2018). Moreover, Bax, Bcl 2, caspase 3, LC3 II activity and other autophagy and apoptosis cytokines were also reversed by curcumin (Hou et al., 2019; Huang et al., 2018; Wang et al., 2005; Xie, C. J. et al., 2018; Xu, L. et al., 2019; Zhang et al., 2018a). Furthermore, curcumin promoted neuron survival *in vitro* to exert neuroprotective effects against ischemia injury (Lu et al., 2018; Xie, W. et al., 2018; Zhang, X. et al., 2018).

### Resveratrol

Resveratrol (Figure 3G), also named 3,4,5-trihydroxy-trans-stilbene, a natural polyphenolic compound, occurs naturally in grapes and a variety of medicinal plants, and possesses multiple biological activities (Santos et al., 2019; Thapa et al., 2019). The effect of resveratrol on cerebral ischemic injury was also explored. Resveratrol dosage at 10–100 mg/kg marked improved neurological deficiency and protected against cerebral ischemic injury though PI3K/Akt, NF -κB and other pathways (Lei et al., 2020; Simao et al., 2012a; Simao et al., 2012a; Yu, P. et al., 2017). Resveratrol treatment could obviously upregulate nuclear factor erythroid 2-related factor 2 (Nrf2) and heme oxygenase-1 (HO-1) to ameliorate oxidative damage in cerebral ischemic injury (Ren et al., 2011; Yang et al., 2018b; Gao et al., 2018). Furthermore, MDA, NO, SOD could be reversed by resveratrol administration (Jie et al., 2020; Tsai et al., 2007; Xu, J. et al., 2018). Silent information regulator 1 (Sirt 1) is a NAD + dependent deacetylase, which plays an important role in cerebral I/R injury (She et al., 2017). It’s reported that resveratrol protected against cerebral ischemic injury by inhibiting NLRP3 inflammasome activation, improving alterations in mitochondrial and glycolytic function through Sirt1 pathway (Della-Morte et al., 2009; Koronowski et al., 2015; He et al., 2017; Koronowski et al., 2017). Moreover, resveratrol could inhibit inflammatory response, including prostaglandin E2 (PGE2), cyclooxygenase (COX)-2, NOS and MMP 9 to alleviate neurological deficits (Candelario-Jalil et al., 2007; Simao et al., 2012b; Wei et al., 2015). In addition, resveratrol could improve brain energy metabolism via inhibiting xanthine oxidase activity and preventing the production of hypoxanthine, xanthine and oxygen radicals (Li et al., 2011). These findings suggested resveratrol could reduce cerebral ischemia injury through multiple pathways.

### Ellagic Acid

Ellagic acid (Figure 3H), an important cell protective and antioxidant compound, is a low molecular weight polyphenol derived from several fruits, vegetables and nuts (De Oliveira, 2016). Ellagic acid are recently more taken into accounts since their promising pharmacological effects, such as, anti-inflammation, anti-oxidant and anti-cancer (Ceci et al., 2018; Baradaran Rahimi et al., 2020). In recent study, ellagic acid is investigated as a potential endowed with multi-target pharmacological properties on central nervous system (Alfei et al., 2019). BCCAO was carried out to make global cerebral ischemic injury. Ellagic acid pretreatment with 100 mg/kg could reduce MDA level and restore the heart rate to normal level (Nejad et al., 2015). In addition, ellagic acid 100 mg/kg could activate PI3K/Akt/NOS pathway to alleviate brain injury and protect brain tissue in MCAO model (Kaihua and Jiyu 2020). Furthermore, ellagic acid administration with 10–90 mg/kg could improve brain injury outcomes and increase the proliferation of NSCs through the Wnt/β-catenin signaling pathway (Liu, Q. S. et al., 2017). The upregulation of zonula occludens-1 (ZO-1) and down-regulation of AQP 4 and MMP-9 in injured brain tissues after being treated ellagic acid 10–50 mg/kg (Wang et al., 2019b).

### Neuroprotective Role of Glycosides in Ischemic Brain Injury

#### Asiaticoside

**FIGURE 4:**
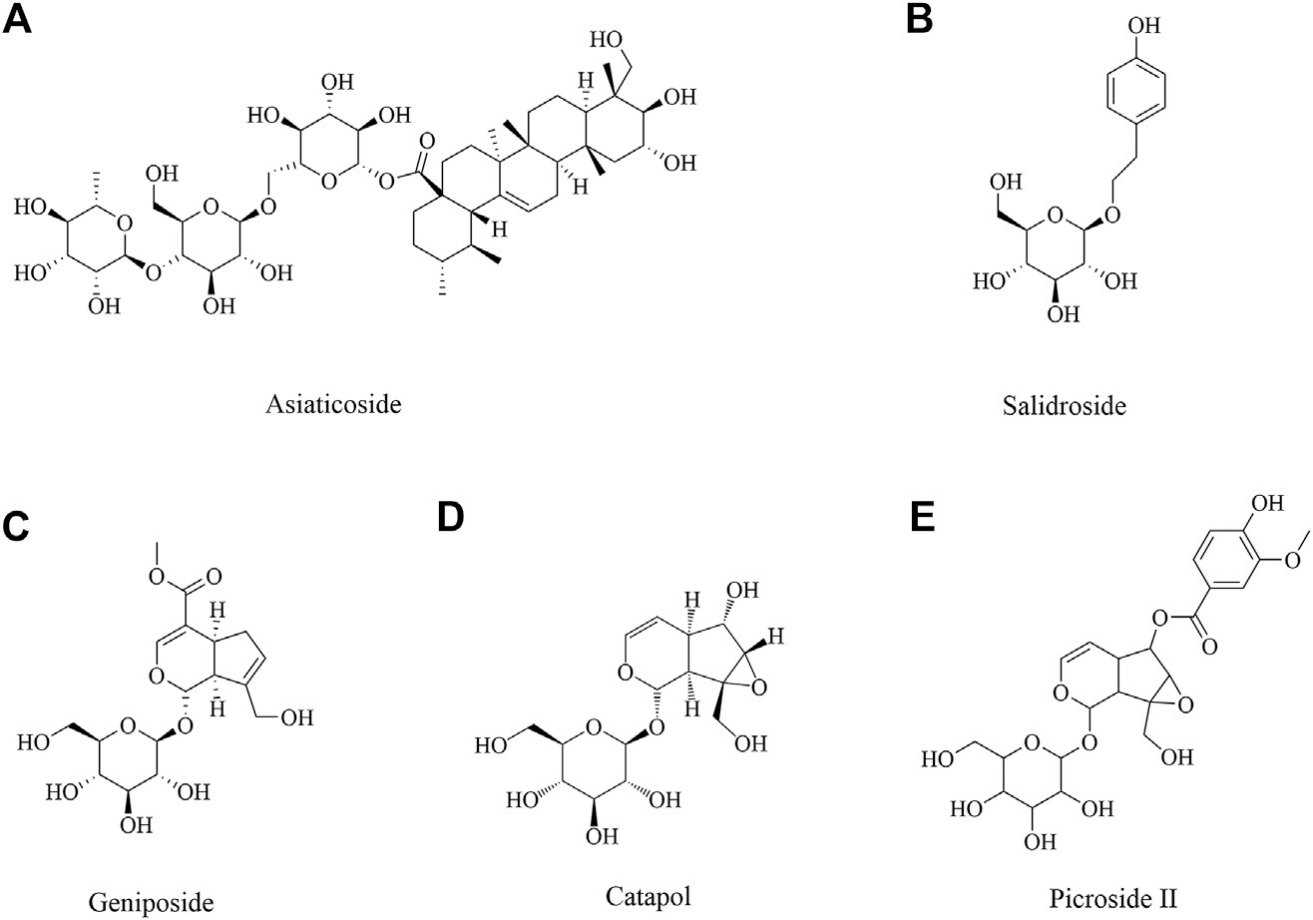
The glucosides for cerebral ischemia injury.

Asiaticoside (Figure 4A) is isolated from *Centella asiatica* (L.), which has been using as a memory enhancing and psychoactive drug for a long time in Asia (Zheng and Qin 2007). Many studies have shown that C. asiatica indeed has many biological activities in central nervous system (Mook-Jung et al., 1999; Zheng and Qin 2007; Dhanasekaran et al., 2009; Orhan 2012). A report demonstrated asiaticoside showed anti-inflammation effect via inhibiting overactivation of p38 MAPK pathway against cerebral I/R mice (Chen et al., 2014). Another study found asiaticoside showed a protective effect against cerebral I/R injury via the NOD2/MAPK/NF-kB signaling pathway (Zhang et al., 2020b).

### Salidroside

Salidroside (Figure 4B), the major phenylpropanoid glycoside extract from medicinal Tibetan plant *Rhodiola rosea* L, has diverse pharmacological activities. Many recent reports and reviews have highlighted that salidroside may exert anti-inflammatory, neuroprotective effects and improve cognitive function (Li, M. et al., 2018; Xu, N. et al., 2019). Salidroside may involve the modulation of monoamine metabolism in the striatum and substantia nigra pars compacta (SNpc), which may be related to the function of the dopaminergic system in the cerebral I/R brain (Zhong et al., 2019). Many studies reported salidroside protected against cerebral I/R injury via PI3K/Akt pathway, accompanying with Nrf2/NF-κB pathway (Wei et al., 2017; Zhang, Y. et al., 2018; Zhang, J. et al., 2019; Zuo et al., 2018). Salidroside exhibited neuroprotective effects against hypoxia/reperfusion injury by activating the SIRT1/FOXO3α pathway (Xu, L. et al., 2018). In addition, salidroside is an effective treatment for ischemic stroke that functions via the fibroblast growth factor 2 (FGF2)-mediated cAMP/PKA/CREB pathway to promote dendritic and synaptic plasticity (Li et al., 2020b). Salidroside treatment reduced the expression of M1 microglia/macrophage markers and increased the exdslbpression of M2 microglia/macrophage markers after stroke and induced primary microglia from M1 phenotype to M2 phenotype (Liu et al., 2018). What’s more, salidroside reduced the markers of endothelial activation and neutrophilic infiltration after I/R injury by inhibition of complement, restoring an anti-inflammatory endothelial phenotype after oxidative stress and inhibiting classical complement activation, in association with anti-apoptotic effects (Wang, Y. et al., 2020).

### Neuroprotective Role of Iridoid Glycosides in Ischemic Brain Injury

Iridoid glycosides are special glycosides. Iridoid glycoside exists broadly in plants of many families and has a wide variety of biological activities including purgative, liver protective, anti-microbial, analgesic, antitumor, sedative and anti-inflammatory activities (Ismailoglu et al., 2002). Geniposide, Catalpol and Picroside II were investigated in depth as iridoid glycosides, which had a neuroprotective effect.

### Geniposide

Geniposide (Figure 4C), as an iridoid glycoside, was initially isolated from the herb *Gardenia jasminoides* Ellis (Wang et al., 1992), which has been noted for its variable pharmacological effects, including anti-oxidation, anti-inflammation, and anti-diabetes effects, among others (Koo et al., 2006; Wu et al., 2009). Previous studies showed that geniposide induced neuronal differentiation and attenuated cell injury (Liu et al., 2009; Yin et al., 2010). To date, there were some studies about the cerebral ischemia injury. Geniposide treatment after neonatal hypoxic-ischemia (HI) insult attenuated cell apoptosis, IgG leakage, microgliosis, astrogliosis, pericytes loss and junction protein degradation, which might be through the activation of PI3K/Akt pathway (Liu, F. et al., 2019). Geniposide attenuated inflammatory response by suppressing P2Y14 receptor and downstream ERK1/2 pathway in brain microvascular endothelial cells (BMECs) with OGD (Huang et al., 2017; Li, Y. et al., 2016). What’s more, geniposide in neuroprotection by activating autophagy and inhibiting NLRP3 inflammasome in microglial cells (Fu et al., 2020).

### Catalpol

Catalpol (Figure 4D) is the main active component of the radix from *Rehmannia glutinosa* Libosch, and it belongs to the iridoid monosaccharide glycoside family (Ismailoglu et al., 2002; Zhang et al., 2008), which has pleiotropic protective effects on many diseases, including neurodegenerative diseases (Xia et al., 2012), ischemic stroke (Zhu et al., 2010), metabolic disorders (Zhu et al., 2010) and others. It’s reported that the efficacy of catalpol pretreatment on cerebral I/R injury may be attributed to reduction of free radicals and inhibition of lipid peroxidation and endothelin-1 (ET-1) production (Liu, H. et al., 2014). Additionally, a study by Li et al. found catalpol also exerted the most significant cytoprotective effect on astrocytes by suppressing the production of free radicals and elevating antioxidant capacity (Li et al., 2008). What’s more, catalpol significantly inhibited apoptosis by modulating Bcl-2 and Bax (Li et al., 2006). Catalpol affected angiogenesis via the JAK2/STAT3 signaling pathway and VEGF expression (Dong, W et al., 2016).

### Picroside II

*Picrorhiza scrophulariflora* belongs to the plant family composed of picroside I, II and III, of which picroside II (Figure 4E) is one of the most effective components extracted from the dried rhizome and roots of *Picrorhiza kurrooa* Royle ex Benth and *Picrorhiza scrophulariae flora* Pennell (Stuppner and Wagner 1989; Wang et al., 1993). Current researches on picroside II are focused on its neuroprotective, anti-apoptotic, anti-cholestatic, anti-oxidant, anti-inflammation, immunomodulating activities (Cao et al., 2007; Li et al., 2007; He et al., 2009). It has been confirmed that picroside II 25 mg/ml could enhance nerve growth factor - induced PC12 cell axon growth, reduce H_2_O_2_ induced PC12 cell damage and improve cell survival *in vitro* (Cao et al., 2007). Picroside II attenuated cerebral I/R injury via inhibiting apoptosis and inflammation, included COX2, TLR4/NF-κB and MEK-ERK1/2 pathway (Guo et al., 2010; Wang, F. et al., 2015; Wang, L. Y. et al., 2015). Picroside II could protect BBB possibly through reducing oxidative stress factors (ROS, NOX2 ROCK, MLCK, and MMP-2) and enhancing BBB function factors, claudin-5 (Zhai et al., 2017). Furthermore, picroside II exerted a neuroprotective effect by inhibiting the mitochondria Cyt C signal pathway and decreasing the permeability of mitochondrial permeability transition pore (mPTP) following I/R injury in rats (Zhang et al., 2017; Li. Q et al., 2018).

### Neuroprotective Role of Saponin in Ischemic Brain Injury

**FIGURE 5:**
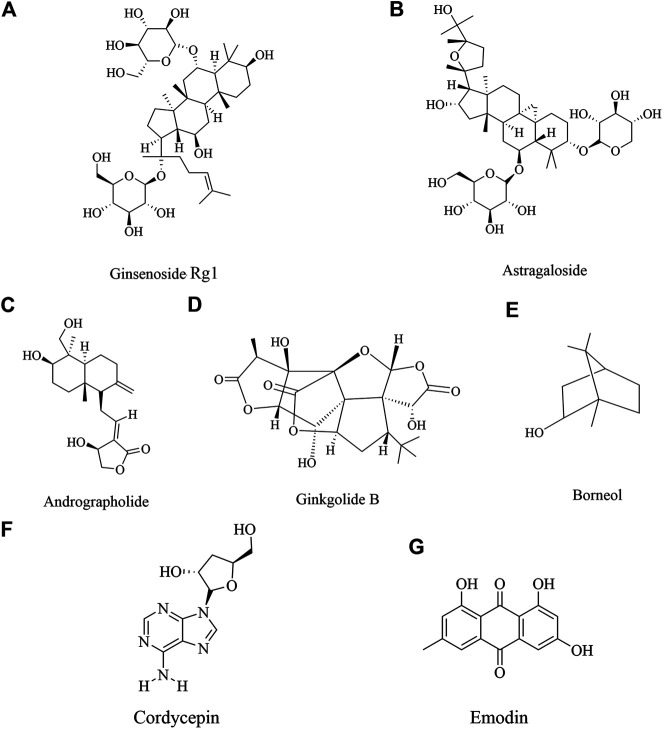
The saponin, terpenoids and others for cerebral ischemia injury.

Ginsenoside Rg1 (Figure 5A) is the representative components in saponin. Ginsenoside Rg1 is one of the main active ingredients of ginseng (Zhang and Zhao 2014; Chuang et al., 2015). It has been shown that as a small molecular substance, ginsenoside Rg1 easily passes through the blood brain barrier. Moreover, ginsenoside Rg1 could promote stem cell orientation transformation, induce stem cell proliferation and played a neuroprotective role in brain repair (Cheng et al., 2005; Tang et al., 2017; Xie, C. J. et al., 2018). It’s reported that ginsenoside Rg1 could relieve the I/R injury through multiple pathways. Ginsenoside Rg1 could improve neurological injury, regulate BBB disruption and permeability and downregulate AQP 4 and protease-activated receptor-1 (PAR 1) (Zhou et al., 2014; Xie et al., 2015). Ginsenoside Rg1 alleviated oxidative stress after I/R through inhibiting miR-144 activity and subsequently promoting the Nrf2/ARE pathway (Chu et al., 2019). What’s more, a study by Li et al. found that ginsenoside Rg1 may exert its neuroprotective action on cerebral I/R injury through the activation of PPARγ signaling (Li et al., 2017). In addition, it’s also shown that ginsenoside Rg1 treatment obviously decreased cell apoptosis, while the transplanted cells could be differentiated into neurons and glial cells, which also improved cerebral ischemia (Bao et al., 2015). Other studies showed that ginsenoside Rg1 40 mg/kg was attributed to a decrease in ubiquitinated aggregates and a suppression of the inflammatory response and increased in the expression of BDNF in the hippocampal CA1 region after I/R insult (Wang, Y. et al., 2018; Zheng et al., 2019).

### Astragaloside IV

Astragaloside IV (Figure 5B) is a triterpenoid saponin existing in the root of *Astragalus membranaceus*. Astragaloside IV also has a variety of pharmacological effects, such as anti-inflammatory, anti-cancer, anti-fibrosis, anti-oxidation stress, immunomodulatory through multiple signals (Ren et al., 2013; Zhang, X. et al., 2019). Meanwhile, it showed that astragaloside IV could inhibit neuroinflammation by reducing BBB permeability and lymphocyte infiltration, and played a neuroprotective role through mitochondrial pathway, antioxidant, anti-inflammatory and anti-apoptotic effects (Costa et al., 2018; Song et al., 2018), which mainly regulated JNK3, FAS, FasL, caspase-8, Bid, caspase-3 and cyto C, p62, Bax/Bcl-2, LC3II/LC3I (Li et al., 2019; Liu et al., 2013; Yin et al., 2020; Zhang, J. et al., 2019). In addition, astragaloside IV could also inhibit neutrophil adhesion related molecules (TNF-a, NF κB, IL-1β, etc.) to play an anti-inflammatory role, and had neuroprotective effect on cerebral I/R injury (Li et al., 2012).

## Neuroprotective Role of Terpenoids in Ischemic Brain Injury

### Andrographolide

Andrographolide (Figure 5C), a labdane diterpene lactone, is the most active and important constituent isolated from the leaves of *Andrographis paniculata* (Burm. f.) Nees (Acanthaceae) (Coon and Ernst 2004). Recent studies demonstrated that andrographolide possesses anticancer, anti-inflammatory and hepatoprotective activities, also neuroprotective effect (Negi et al., 2008; Bao et al., 2009). Andrographolide reduced NOX2 and iNOS expression possibly by modulating PI3K/AKT-dependent NF- κB and HIF-1 α activation, which mediated the protective effect in the cerebral I/R mice (Chern et al., 2011). Studies by Yen et al. found andrographolide could play an important role to cerebral endothelial cells (CECs). Furthermore, andrographolide increased Nrf2/HO-1 expression through p38 MAPK regulation, which provided protection against I/R injury (Yen et al., 2013; Yen et al., 2016).

### Ginkgolides

Ginkgolide B (Figure 5D) were one of ten kinds of diterpene lactone compounds, which were isolated from *Ginkgo biloba* leaves. It’s reported that exact of *Ginkgo biloba* had a broad range of pharmacological effects, such as anti-inflammation, antioxidant, anti-depressant (Chen, C et al., 2017; Hu et al., 2018; Jin, G et al., 2014; Ran et al., 2014; Saini et al., 2014; Wan et al., 2016). Ginkgolides B upregulated the levels of antioxidant proteins through Akt/Nrf2 pathway to protect neurons from oxidative stress injury (Liu, Q. et al., 2019). In addition, ginkgolide B improved neurological function by promoting the proliferation and differentiation of neural stem cells in rats with cerebral I/R injury (Zheng et al., 2018b).

### Borneol

Borneol (Figure 5E) is a terpene and bicyclic organic compound, a resin from *Cinnamomum camphora* (L.) Presl*,* a traditional Chinese medicine (Yin et al., 2017; Zheng et al., 2018a). As a traditional Chinese medicine, borneol has been used for thousands of years in the treatment of cardiovascular and cerebrovascular diseases. Modern pharmacological studies have shown that borneol had anti-inflammatory, antioxidant stress, anti-apoptosis and other pharmacological effects (Almeida et al., 2013). Borneol protected against cerebral I/R injury through multifunctional cytoprotective pathways, involving in the alleviation of intracellular ROS and iNOS/NO pathway, inhibition of inflammatory factor and depression of caspase-related apoptosis. Additionally, the inhibition of IκBα/NF-κB pathway might play a significant role in the neuroprotection of borneol (Liu et al., 2011). Another study by Dong et al. found that borneol could alleviate cerebral ischemic injury, most likely executed via anti-apoptosis and anti-inflammation effects and maintenance of the BBB stability and TJs to comprehensively improve NVU function (Dong et al., 2018). Additionally, the protection of OGD induced BMECs by tetramethylpyrazine phosphate and borneol combination involved anti-oxidation, apoptosis inhibition, and angiogenesis (Yu et al., 2019).

## Neuroprotective Role of Other Compounds in Ischemic Brain Injury

### Emodin

Emodin (Figure 5F), 1,3,8-trihydroxy-6-methylanthraquinone, is a naturally occurring anthraquinone derivative and an active component from *Polygonum multiflorum* Thunb. *Rheum palmatum* L. etc, which have been used widely in Asia in treatment of multiple diseases (Dong, X. et al., 2016). Emodin has been demonstrated to possess a wide spectrum of pharmacological effects, such as anti-viral, anti-bacterial, anti-allergic, anti-osteoporotic, immunosuppressive, neuroprotective activities (Dong, W. et al., 2016; Leung et al., 2020; Xue et al., 2020). In fact, the neuroprotective effect of *Polygonum multiflorum* Thunb was first published in 2000 (Gu et al., 2000) and the neuroprotective effect of emodin was published in 2005 when its ability to interfere with the release of glutamate was identified as a method of neuroprotection (Gu et al., 2005). Additionally, emodin might afford a significant neuroprotective effect against glutamate-induced apoptosis through the critical role including Bcl-2/Bax, active caspase-3, p-Akt, p-CREB, and mature BDNF for potent neuroprotective effects of emodin to subsequently enhance behavioral function in cerebral ischemia (Ahn et al., 2016). Another study by Leung et al. found emodin had neuroprotective effects against I/R or OGD injury both *in vitro* and *in vivo*, which may be increase Bcl-2 and glutamate transporter-1 (GLT-l) expression but suppress activated-caspase 3 levels through activating ERK1/2 pathway (Leung et al., 2020).

### Cordycepin

Cordycepin (Figure 5G) is a derivative of nucleoside adenosine and one of the main active components of *Cordyceps sinensis*, which is mainly distributed in Europe, North America and Asia (Tuli et al., 2013; Wang, Y. et al., 2015). Cordycepin has a variety of pharmacological activities, such as anti-inflammatory, anti-tumor, antioxidant, anti-inflammatory microorganism, anti-hyperlipidemia, anti-hepatotoxicity, anti-depressant and neuroprotective activities (Ryu et al., 2014; Wang et al., 2015a; Qin et al., 2019). It was shown that cordycepin had an important neuroprotective effect on hypoxic injury by improving the electrophysiological function of neurons (Chen, K. et al., 2017; Liu, Z. B et althogenesis of lesions (Liu, G. et al., 2015). It is confirmed that cordycepin could significantly reduce glutamic acid and aspartic acid, increase SOD activity and down-regulate MDA and MMP-3 to alleviate cerebral ischemic injury or hypoxia injury, suggesting cordycepin could inhibit oxidative damage to exert neuroprotection (Hwang et al., 2008; Cheng et al., 2011). Cordycepin regulated adenosine A1 receptor to improve long-term potentiation formation and neuronal survival through p38/JNK/ERK pathway in BCCAO model and glutamate-induced HT22 neuronal cell death (Dong et al., 2019; Jin, M. L et al., 2014).

### Polysaccharides

Polysaccharides are considered to have a wide range of pharmacological effects, such as scavenging free radicals, immune regulation, anti-tumor, anti-oxidation, anti-viral, anti-inflammatory, lowering blood sugar, anti-depression, liver protection, etc (Jin et al., 2012; Kwok et al., 2019; Fang et al., 2020). Panax notoginseng polysaccharide is a kind of heteroglycan derived from the medicinal plant *Panax notoginseng*, which could increase the ratio of Bcl-2/Bax and reduce caspase-3 in cerebral ischemic brain tissue (Jia et al., 2014). What’s more, it could enhance GSH-Px, SOD activity and IL 10 level, while downregulate MDA, TNF-α, IL-1β level to reduce cerebral infarction size and cell apoptosis to afford neuroprotective effect (Jia et al., 2014; Sy et al., 2015). Angelica polysaccharide is the main active ingredient of *Angelica sinensis* (Oliv.) Diels, which could also enhance the activities of SOD, GSH and GSH-PX, and reduce MDA, IL-1β, TNF-α and NF-κB in cerebral ischemia-reperfusion injury rats (Yan and Xie, 2018; Dai et al., 2020), in addition, enhance angiopoietin 1, angiopoietin 2, VEGF to promote angiogenesis (Hu et al., 2006; Li, J. et al., 2019). Ginkgo biloba polysaccharides could also play a neuroprotective effect by inhibiting oxidative stress and inflammation to improve neurological deficits in cerebral ischemic animals (Yang et al., 2013). Black fungus polysaccharide is the main active ingredient of *Auricularia auricula* (L.ex Hook.) Underw in China. Studies have found that fungus polysaccharides could reduce the production of ROS, MDA, and NO in rats with acute or chronic cerebral ischemia injury, enhance the activity of SOD and played an anti-oxidant effect. In addition, it can inhibit neuronal apoptosis and improve cerebral ischemia-reperfusion injury. (Lu et al., 2010; Qian and Min 2010; Ye et al., 2010). Fucose is a marine sulfated polysaccharide extracted from brown algae and some marine invertebrates (Apostolova et al., 2020; Oliveira et al., 2020). Experimental studies have shown that fucose can significantly reduce the levels of pro-inflammatory factors IL-1β, IL-6, MPO and TNF-α in cerebral ischemia model animals, reduce the levels of oxidative stress-related proteins SOD and MDA, and could also regulate cell apoptosis via inhibiting the MAPK pathway to play a neuroprotective effect in cerebral ischemia reperfusion study (Che et al., 2017). Other studies have shown that fucose could also play a neuroprotective effect by inhibiting neuronal apoptosis, glial cell activation and anti-oxidative stress properties (Ahn et al., 2019; Kim et al., 2019). Codonopsis pilosula polysaccharide is a kind of polysaccharide extracted from Codonopsis pilosula. Studies have shown that Codonopsis pilosula polysaccharide could reduce LDH, NO and MDA, increase the content of AChE, SOD and GSH-Px, and inhibit the expression of Beclin-1 to regulate autophagy in the cerebral ischemia-reperfusion injury model (Chen, W. et al., 2015; Ma et al., 2019). It could also inhibit the expression of GFAP protein (Li. S. et al., 2018). Codonopsis pilosula polysaccharides can also reduce the expression of Bax, up-regulate the expression of Bcl-2 and inhibit apoptosis to exert neuroprotective effects through mediating the Nrf2/HO-1 pathway (Ma et al., 2019). Lycium barbarum polysaccharide, astragalus polysaccharide, aloe polysaccharide, yam polysaccharide, etc. also have a certain protective effect on cerebral ischemia (Ge et al., 2017; Liu, H. et al., 2014; Lu et al., 2012; Peng et al., 2019; Wang, Y. et al., 2014; Yan and Zhou, 2012).

### Structure-Activity Relationship Between Natural Products and Neuroprotection

**FIGURE 6:**
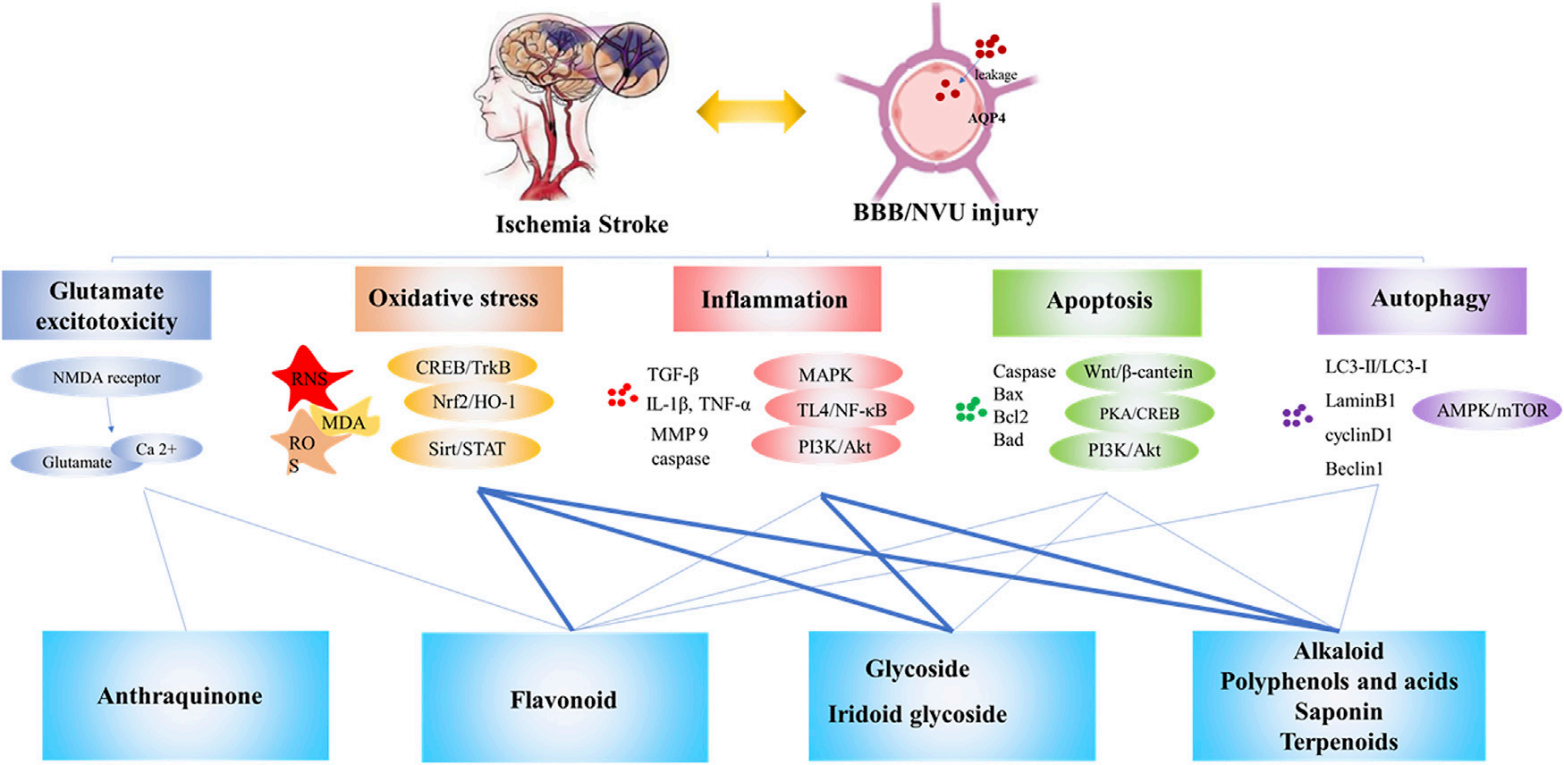
The mechanism of cerebral ischemia and the neuroprotective effect of natural products against cerebral ischemia. Note: Bold lines indicate a clear structure-activity relationship reported in the literature.

Many studies have confirmed the neuroprotective effect of natural products on cerebral ischemia and clarified its preliminary mechanism of action. Different natural products have different mechanisms of anti-cerebral ischemia, which are closely related to the different structures of the compounds. Figure 6 shows the mechanism of cerebral ischemia and the role of different types of natural products in this process. The research on the structure-activity relationship of the biological activity of natural products is mainly related to the basic nucleus of the compound, the number and position of double bonds, and the position of functional groups. They have a great influence on the biological activity of the compound (Aljahdali et al., 2020; Zhou et al., 2021). The flavonoids are based on 2-phenylchromone as the skeleton, which has ortho-dihydroxyl in the structure. The hydroxyl substituent on the basic skeleton is the key functional group for scavenging oxygen free radicals. At the same time, a conjugated system is also the key active site to scavenging oxygen free radicals (Chen et al., 2013). Therefore, flavonoids have unique advantages in scavenging free radicals and inhibiting lipid peroxidation. In addition, flavonoids had anti-inflammatory effects. It is believed that when the C-5 and 7 positions of the A ring of flavonoids have hydroxyl groups at the same time, they will affect the secretion process, mitosis and cell interactions of cells, and then produce strong anti-inflammatory effect (Ying and Chi, 2007).

Alkaloids are quite different in structure. For example, berberine has a methylenedioxy ring structure and is the main functional group for anti-bacterial and hypoglycemic activity (Han et al., 2020; Wu and Fang, 2020). This may also be the key structure of its antioxidant effect, which needs further research to confirm. Ligustrazine has a simple structure and a wide range of pharmacological effects. Use it as a lead compound to synthesize various derivatives, which has a variety of biological activities (Wang et al., 2013), and the derivative is more stable than ligustrazine, such as ligustrazine hydrochloride injection has better curative effect in the treatment of ischemic cerebrovascular disease (Li and Zhang, 2020; You et al., 2020). Dauricine is a kind of bisbenzyltetrahydro isoquinoline alkaloid. It has a phenolic hydroxyl group and a conjugated system that is easy to form hydrogen bonds. It may be similar to flavonoids and is considered to be a key structure for antioxidant effects. Studies have shown that phenolic acids have a strong antioxidant effect, which is due to the existence of carboxyl, carbonyl and other structures (Bialecka-Florjanczyk et al., 2018; Wang, Y. et al., 2019). Glycoside components are compounds formed by non-sugar substances and sugars or sugar derivatives, which have the active characteristics both sugar substances and non-sugar substances. For example, flavonoid glycosides and flavonoid aglycones have anti-oxidative biological activity (Xue et al., 2001; Gao et al., 2008; Shao et al., 2013). In addition, most studies believe that glycoside compounds mainly exerted neuroprotective effects by improving the body’s immunity (Brito-Arias, 2007). Iridoid glycosides are a special glycoside chemical. The core of iridoid glycosides is hemiacetal-enether cyclopentane, which is an important structural basis. Anti-oxidant stress is the manifestation of its biological activity (Wang et al., 2019a). Some studies also believe that the double bond on the cyclopentane in iridoid glycosides, the C-11 substituent, and the way of forming the bond after ring opening also have important effects on the anti-inflammatory activity (Mao et al., 2019). For example, loganin, morroniside, and sorgenin all contain several electron-donating groups-hydroxyl groups. The hydroxyl groups release hydrogen atoms and combine with free radicals to block the chain reaction mediated by reactive oxygen species and reduce the body's oxidative stress. Stimulus levels, which weaken the phosphorylation of inflammatory signal pathway related factors, thereby inhibiting the inflammatory response, improving insulin resistance and liver damage (Li et al., 2014). Studies believe that tetracyclic triterpene saponins are the leading compounds for the treatment of neurological diseases, and can show strong neurological activity after proper structural modification and modification. Among them, C-7 and C-9 of lanolin triterpenoids The diene bond connected at the position may be the key group for nerve extension promoting activity (Liu, Y. et al., 2015). The research on the structure-activity relationship of tetracyclic triterpenes is mainly focused on the study of the binding sites related to the action of NMDAR antagonists, indicating that the structural modification and optimization of its aglycon and side chain sugar groups can obtain a new type of neuroactive monomer with high efficiency and low toxicity drug. Tetracyclic triterpene dammarane compounds as NMDAR antagonists will be an effective way to treat neurological diseases (Lee et al., 2006; Ryoo et al., 2020). Studies believe that ginkgolides can improve cerebral blood circulation and energy metabolism through specific inhibition of PAFR, and show a good market prospect in the prevention and treatment of cardiovascular and cerebrovascular diseases. The main differences in their effects are: the number and positions of hydroxyl groups contained in ginkgolides are different, PAF and ginkgolide ligands can be combined, and the introduction of benzyl derivatives at position C-10 is more beneficial for antagonistic activity; In addition, the presence of C, D ring and tert-butyl group also plays an important role in activity (Hui et al., 2013). Some scholars have studied the structure-activity relationship of andrographolide and found that the position of the double bond determines the anti-inflammatory effect of andrographolide and its derivatives, and the anti-inflammatory effects of compounds with double bonds in the ring are stronger than those with double bonds outside the ring (Tian et al., 2015). As a natural component of polycarbonyl and polyhydroxyl groups, emodin can coordinate and chelate with the zinc ions of type IV collagenase to inactivate type IV collagenase, thereby preventing type IV collagenase (MMP-2, MMP- 9) degradation of basement membrane and extracellular matrix of normal tissue cells, play a role in anti-invasion and anti-metastasis (Wang, 2006). As a nucleoside antibiotic, cordycepin has a good application prospect in regulating immunity and scavenging free radicals (Lee et al., 2020).

In summary, the activity of natural products has an important relationship with the structure of the substance itself. The active functional groups of natural products are the key to their functions. Moreover, the bioavailability of many natural products is very low, which limits their use, the structural modifications, changes in dosage forms or improvements in pharmacokinetic parameters can improve their bioavailability. Because the pathogenesis of cerebral ischemia is complex, and cascade reactions occur cross-over. The anti-cerebral ischemia effects of individual natural products are often weak. Therefore, a full understanding of the mechanism of the cerebral ischemia cascade can provide the possibility for the development of specific mechanisms or targeted compounds, and the combined use of different natural products to play a multi-target neuroprotective effect may be a promising therapy. However, it is very important to combine animal models with clinical practice (Ma et al., 2020). Choose appropriate experimental models according to actual conditions to provide a solid foundation for the clinical transformation of natural products.

## Conclusions and Outlook

Stroke is one of the most common causes of disability and death worldwide, seriously endangers human health and brings heavy burden to society and family. Till now, there is no effective therapy which is available for the treatment of cerebral ischemia. Recombinant plasminogen activator is the only medicament utilized clinically and its use is restricted due to short therapy time windows and the risk of bleeding. Natural products from plants have been used to treat the cure of numerous disorders through the previous practice of therapeutics (Fan et al., 2020; Li Y. et al., 2017; Savopoulos et al., 2009; Chen, S. et al., 2020). Nowadays, many attempts have been made to explore the neuroprotective effects of natural products with the advance of technology. Hence, this review summarizes recent studies on the biological activities and mechanisms of recognized compounds for cerebral ischemia injury prevention or/and treatment (Table 1). Through the literatures, three aspects should be noted with special focus. First, multiple types of natural products, including flavonoids, alkaloids, saponin, terpene, iridoid glycosides, polyphenols and others, are proven to have a definite effect on cerebral ischemia injury. In fact, the clinical efficacy still requires further confirmation and to study. Second, animal models and cell experiments were used to investigate the neuroprotective effect of natural product on cerebral ischemia injury. Animal models including MCAO, BCCAO and other occlusion methods were used to induce cerebral ischemia injury. Due to the diversity of brain cells, a variety of cell lines are used in the study of neurological diseases, for example, HT22, BV2, BMEC and others. Due to the limitations of cell models, the neuroprotective effects of many natural products in cell models still need to be verified by *in vivo* experiments. Third, most of the compounds show neuroprotective effects through multiple targets or signaling pathways. These natural products work by comprehensive regulation, inhibiting excitotoxicity, influencing free radicals, anti-apoptosis, impairing blood-brain barrier disruption, anti-inflammation, influencing astrocytic activation and proliferation. The mechanism by which natural products has multiple targets and diverse signaling pathways. Therefore, natural products would be very valuable when seeking novel therapeutic agents for stroke.

**TABLE 1:** Summary a few natural products and their targets of action imparting neuroprotective activity.

Categories	Natural products	Dosage	Targets/pathway	References
Flavonoid	Baicalin	50, 100, 200 mg/kg (*in vivo*), 10 μg/ml (*in vitro*)	MDA, SOD, GSH, GSH-Px, caspase-3, BDNF, NOD2, TNFα, IL-1β, COX-2, iNOS, NO, PGE2, TLR2/4, NF-κB p65, MCL-1, Bcl-2 MRTF-A, PI3K, ERK1/2	(Cao et al., 2011; Li, F et al., 2010; Tu et al., 2011; Xue et al., 2010; Zheng et al., 2015)
	Scutellarin/scutellarein	50, 100 mg/kg (*in vivo*), 0.54 mM (*in vitro*)	TNF-α, IL-1β, JNK, ERK, p38, iNOS, eNOS, nNOS, ROS, NOX2, Cx43, SOD, MDA, NO, caspase-3, Notch-1, NICD, HES-1, VEGF, bFGF, ACE, ANG II, AT1R, IL-6	(Chen, S et al., 2020; Fang et al., 2016; Fang et al., 2015; Hu et al., 2005; Sun, R et al., 2018; Tang et al., 2014; Wang et al., 2016)
	Apigenin/Vitexin	2–40 mg/kg (*in vivo*), 2.5, 5, 10μM and 0.5, 2.5, 10 nM (*in vitro*)	IL-1β, IL-6, TNF-α, IL-10, LDH, SOD, MDA, NO, eNOS, iNOS, bax, Bcl-2, Caspase-3, PARP, MMP-2, MMP-9, ROCK2, RhoA, VEGF, BDNF, PI3K/Akt, ERK, JNK, p38, mTOR, PPAR-γ, Caveolin-1, Beclin1, p62, LC3Ⅰ, LC3Ⅱ, NKCC1, Ulk1, Keap1, HO-1, Nrf2, CREB	(Cui et al., 2019; Jiang et al., 2018; Pang et al., 2018; Tu et al., 2017; Wang et al., 2015b; Zhang, X. et al., 2019)
	Icariin	10, 30 mg/kg (*in vivo*), 0.25, 0.5, 1 mg/L (*in vitro*)	IL-1 β, IL-6, TNF-α, TGF-β1, NF-κB p65, PPARα, PPARγ, IκB-α, IRE1α, XBP1u, XBP1s, caspase-3, Bcl-2, bax	(Deng et al., 2016; Xiong et al., 2016; Mo et al., 2020; Yang et al., 2020)
	Quercetin	5–25 mg/kg (*in vivo*), 10 μM (*in vitro*)	TNF-α, IL-1β, LDH, ERK, akt, EGF, MMP-9, Claudin-5, ZO-1, β-catenin, GSK-3β, Axin, LEF1, MDA, SOD, CAT, caspase-3, Bcl-2, Nrf2, NOX4, IκBα, p65	(Dai et al., 2018; Jin et al., 2019; Park et al., 2020; Wang, Y et al., 2020)
	Calycosin	5–30 mg/kg	BDNF/TrkB, TNF-α, Bcl-2, NBR1, p62, caveolin-1, claudin-5, NO, ZO-1, MMP-2, MMP-9, ROS, ER-α, RASD1, Bcl-2, SIRT1, FOXO1, Bcl-2, bax, PGC-1α	(Guo et al., 2012; Hsu et al., 2020; Wang, Y et al., 2014; Wang, Y. et al., 2018)
Alkaloid	Berberine	0.002–100 mg/kg (*in vivo*), 0.5 μg/ml (*in vitro*)	PI3K/Akt, p53, cyclin D1, caspase 3, bad, p55γ, BDNF, TrkB, GSK3β, CREB, claudin-5, NF-κB, HIF-1α	(Hu et al., 2012; Song et al., 2012; Zhang et al., 2012; Chai et al., 2013; Zhang et al., 2016b; Yang et al., 2018b)
	Ligustrazine	10–100 mg/kg (*in vivo*), 1, 10, 100 mM (*in vitro*)	IL-1β, IL-6, TNF-α, IL-10, SOD, GSH-Px, MDA, p53, Caspase-3, Bcl-2, bax, mTOR, ULK1, BNIP3, Beclin1, LC3 II/I, Bax/Bcl-2, HIF-1α, MPO, Nrf2, HO-1, ERK, IFN-γ、TLR4、HMGB1, occludin, JAM-1、AQP4, MMP9, NO, iNOS, eNOS, akt, MCP-1, ICAM-1, TLR-4/NF-κb p65	(Chang et al., 2015; Chang et al., 2007; Ding et al., 2019; Xu et al., 2017; Yan et al., 2015; Yu, P. et al., 2017; Yu et al., 2016; Zhou et al., 2019)
	Daurisoline	5, 10, 20 mg/kg	Cyt-C, Caspase 3, Caspase 9, SOD, MDA, GSH-Px	(Li and Gong 2004; Yang et al., 2009; Lan and Lianjun 2020)
	Tetrahydropalmatine	10, 20, 40 mg/kg	MPO, NO, ONOO2-, iNOS, p85, eNOS, akt, HIF-1, VEGF, TNF-α, occludin, ZO-1, claudin-5, caveolin-1, MMP-2/9, src, MLCK, p-MLC, p38, bax, caspase-3, Bcl-2, PRAP	(Han et al., 2012; Mao et al., 2015; Sun, R et al., 2018)
	Peimine	1–5 mg/kg	SOD, MDA, LDH, IL-6, IL-10, IL-18, ICAM-1, IL-1β, caspase-9, caspase-3, bax, bcl-2, LC3Ⅱ/LC3Ⅰ, beclin1, p62, PI3K、Akt, mTOR, TNF -α, β-arrestin	(Guo et al., 2019; Duan et al., 2020)
Polyphenols and acids	Curcumin	30–300 mg/kg (*in vivo*), 1.25–20 μM (*in vitro*)	MDA, GSH, GSH-Px, SOD, NF-κB p65, Nrf2, IL-1β, IL-8, JAK2, STAT3, akt, mTOR, LC3-II, LC3-I, p62, TLR4, p-38, IL-1, IL-6, TNF-α, iNOS, caspase-3, bax, Bcl-2, Bcl-XL, COX-2, Nrf2, NO, HO-1, MEK, ERK, CREB, LDH, MnSOD, AIF, caspase-9/-3, Trx-2, MPT, MMP-9, ZO1, occludin, HIF-1α, JNK	(Huang et al., 2018; Li et al., 2015; Li et al., 2016; Lu et al., 2018; Xie, C. J. et al., 2018; Xu, N. et al., 2019)
	Resveratrol	20–40 mg/kg (*in vivo*), 1–50 µM (*in vitro*)	NF-κb p65, NO, iNOS, eNOS, nNOS, JNK, GFAP, PI3K/Akt, GSK-3β, CREB, PGE, COX-1, COX-2, LDH, Bcl-2, Caspase-3, NQO-1, Sirt1, UCP2, LC3B-II/I, p62, NLRP3, caspase-1, IL-18, Nrf2/HO-1, Caspase-3, GSH-Px, SOD, CAT, MDA, TNF-a, IL-1β, IL-6, LDH, MMP-9, TIMP-1, shh	(Candelario-Jalil et al., 2007; Simao et al., 2012b; Wei et al., 2015; He et al., 2017; Koronowski et al., 2017; Yang et al., 2018b)
	Ellagic acid	10–100 mg/kg	Bax, bcl 2, cyt C, caspase 3, PI3K/Akt, NOS, MDA, wnt/β-catenin, ZO-1, AQP 4, MMP-9	(Kaihua and Jiyu 2020; Liu, Z. B. et al., 2017; Nejad et al., 2015; Wang, F. et al., 2019)
Glycoside	Asiaticoside	20, 40, 60 mg/kg	TNF- α, IL-6, IL-1β, MCP1, ROS, MDA, LDH, SOD, bcl 2, bax, caspase3, NOD2, p38 MAPK, NF-kB p65, JNK, ERK, IκBα	(Chen et al., 2014; Zhang et al., 2020b)
	Salidroside	25, 50, 100 mg/kg (*in vivo*), 1, 10, 100 µM (*in vitro*)	Caspase-3, Bcl-2, bax, ROS, iNOS, LDH, PARP, FGF2, cAMP/PKA/CREB, TNF-α, IL-1β, IL-6, Arg1, TGFβ, IL-2, IL-8, akt, HIF-1α, HIF- 2α, HIF- 3α, EPO, nrf2/HO-1, NF-κB p50, PI3K/PKB, cyt-c, MMP9, Claudin 5, occludin	(Li et al., 2020; Liu et al., 2018; Wei et al., 2017; Zhang, Y. et al., 2018; Zhang X. et al., 2019; Zhong et al., 2019; Zuo et al., 2018)
Iridoid glycoside	Geniposide	5, 10, 20 mg/kg	LDH, TNF-α, IL-1β, IL-6, IL-8, IL-18, NLRP3, ASC, caspase-1, raf/mek1/2/erk1/2, MCP-1, ZO-1, occludin, Claudin-5, β-catenin, PI3K/Akt, Bcl-2/Bax, AchE, NOS, MDA, SOD	(Fu et al., 2020; Huang et al., 2017; Li, W. et al., 2016; Liu, Q et al., 2019)
	Catalpol	5, 10, 20 mg/kg (*in vivo*) 0.3 mM, 2.8 mM, 27.6 mM, 275.9 mM (*in vitro*)	ET-1, SOD, MDA, CGRP, EPO, STAT3, VEGF, JAK2/STAT3, bax, Bcl-2, ROS, NO, iNOS, GSH-Px, GSH	(Dong, X. et al., 2016; Li et al., 2006; Li et al., 2008; Liu, Y. R et al., 2014)
	Picroside II	10, 20 mg/kg	TLR4, TNF-α, NF-κB p65, ERK1/2, ROS, NOX2, Rac-1 ROCK, MLCK, MMP-2, claudin-5, MEK/erk1/2-cox2, ROS, caspase 3, cyt C	(Guo et al., 2010; Li, S. et al., 2018; Wang, C. et al., 2015; Zhai et al., 2017; Zhang et al., 2017)
Saponin	Ginsenoside Rg1	10, 20, 40 mg/kg	PAR-1, akt, nrf2/HO-1, pparγ/HO-1, ERK, JNK, caspase-3/rock1/mlc, IL-1β, IL-6, TNF-α, IκB, NF-κB p65, AQP4, p38 MAPK, MPO, SOD, CAT, HMGB1, Nrf2/ARE, AMPK/mTOR, AMP/AMPK-GLUT	(Li, S. et al., 2017; Xie, W. et al., 2018; Zheng et al., 2019; Zhou et al., 2014)
	Astragaloside IV	10–50 mg/kg (*in vivo*), 0.1–10 μM (*in vitro*)	STAT-3/TNF-α/il-1β, JNK3, MPO/TNF-α/il-1β, NF-κB, akt, LC3II/LC3I, p62, Fas, FasL, Caspase-8, bax, Bcl-2, bid, cyt C, Caspase-3, PI3K/Akt, ICAM-1	(Li et al., 2012; Li, J et al., 2019; Yin et al., 2020)
Terpenoids	Andrographolide	5 mg/kg	LDH, caspase-3, ERK1/2, p38 MAPK, JNK1/2, nrf2/HO-1, Lamin B1	(Chern et al., 2011; Yen et al., 2013; Yen et al., 2016)
	Ginkgolide B	1, 2, 4 mg/kg	LDH, IL-1β, TNF-α, TLR4, NF-κB, nrf2/HO-1, NQO1, SOD, akt, BDNF, EGF, NGF	(Liu, Q et al., 2019; Zheng et al., 2018b)
	Borneol	200 mg/kg	Bax, Bcl-2, Claudin-5, VEGF, TNF-α, ROS, NO, iNOS, caspase-3, caspase-9, ICAM-1, NF-κB p65	(Liu et al., 2011; Dong et al., 2018)
Others	Emodin	15, 50 mg/kg	ERK-1/2, GLT-1, caspase-3, ERK-1/2, LDH, Bcl-2, bax, akt, CREB, BDNF, TNF-α, IL-1β, IL-6, NF-κB p65, IκBα	(Ahn et al., 2016; Leung et al., 2020)
	Cordycepin	5, 10, 20 mg/kg (*in vivo*), 5, 10, 20, 40, 80 µM (*in vitro*)	Bcl-2, bax, Caspase-3, p53, MAPK	(Chen, K. et al., 2017; Cheng et al., 2011; Dong et al., 2019; Hwang et al., 2008; Jin, G. et al., 2014)
	Panax notoginseng polysaccharide	100, 300 mg/kg	Bcl-2/Bax, caspase-3, GSH-Px, SOD, IL 10, MDA, TNF-α, IL-1β	(Jia et al., 2014; Sy et al., 2015)
	Angelica polysaccharide	30, 45, 60 mg/kg	SOD, GSH, GSH-PX, MDA, IL-1β, TNF-α, NF-κB	(Dai et al., 2020; Yan and Xie, 2018; Hu et al., 2006; Li, Y. et al., 2019)
	Ginkgo biloba polysaccharides	100, 200, 400 mg/kg	SOD, GSH, GSH-PX, MDA, IL-1β, TNF-α	(Yang et al., 2013)
	Black fungus polysaccharide	50, 100 mg/kg	ROS, MDA, NO; SOD	(Lu et al., 2010; Qian and Min, 2010; Ye et al., 2010)
	Fucose	80, 160 mg/kg	MAPK; SOD; MDA; IL-1β, IL-6, MPO; TNF-α	(Che et al., 2017; Ahn et al., 2019; Kim et al., 2019)
	Codonopsis pilosula polysaccharide	1, 2 g/kg	Nrf2/HO-1; Bcl-2; bax; bax; AChE, SOD; GSH-Px	(Chen, J. et al., 2015; Ma et al., 2019; Li et al., 2018)

In conclusion, this review summarizes that the facts are comprehensive and deeply informative about the neuroprotective activities of natural products *in vitro* and *in vivo* experiment. For prophylactic and therapeutic management of stroke, they are promising candidates. Therefore, this review would provide as a reference for current advances in the study on natural products for neuroprotection.

## Abbreviations

t-PA, tissue plasminogen activator; BBB, blood-brain barrier; NVU, neurovascular unit; CNKI, China National Knowledge Internet; FDA Food and Drug Administration; Mcl 1, myeloid cell leukemia 1; Bcl 2, B-cell lymphoma-2; SOD, superoxide dismutase, MDA, malondialdehyde; MCAO, middle cerebral artery occlusion; NF-κB, nuclear factor kappa B; TLR2/4, toll like receptor 2/4; NOD2, nucleotide-binding oligomerization domain protein 2; TNF α, tumor necrosis factor α; MAPK, mitogen-activated protein kinase; p-ERK1/2, phosphorylation extracellular regulated kinase 1/2; VEGF, vascular endothelial growth factor; bFGF, basic fibroblast growth factor, NOX2, nicotinamide adenine dinucleotide phosphate oxidase 2; BCCAO, bilateral common carotid arteries; iNOS, inducible nitric oxide synthase; eNOS, endothelial nitric oxide synthase; mTOR, mechanistic target of rapamycin; p-JNK, phosphorylation c-Jun N-terminal kinase; ACE, angiotensin-converting enzyme; AT1R, Ang II type 1 receptor, TNF-α, tumor necrosis factor -α; IL-6, interleukin-6; IL-1β, interleukin-1β; PARP, poly (ADPribose) polymerase; NAD, nicotinamide adenine dinucleotide; OGD, oxygen–glucose deprivation; I/R, ischemic reperfusion; HBMVECs, human brain microvessel endothelial cells; IκB, inhibitory κB; PPARα, peroxisome proliferator-activated receptor α; PPARγ, peroxisome proliferatoractivated receptor γ; IRE1, inositol requiring enzyme 1; XBP1 X-Box Binding Protein 1; BDNF, brain-derived neurotrophic factor; RASD1, dexamethasone-induced Ras-related protein 1; ER, estrogen receptor; p Akt, phospho-Akt; GSK 3β, glycogensynthase kinase 3-β; CREB, cAMP response element binding protein; HIF-1α, hypoxia inducible factor-1-alpha; GSH-Px, glutathione peroxidase; TJ, tight junction; MMP 9, matrix metalloprotein 9; AQP4, aquaporin 4; Cyt-C, cytochrome C; Sirt 1, silent information regulator 1; HI, hypoxic-ischemia; PAR 1, protease-activated receptor-1; SNpc, substantia nigra pars compacta; PGE2, prostaglandin E2; COX-2, cyclooxygenase; GSH, glutathione; STAT, signal transducer and activator of transcription; Nrf2, nuclear factor erythroid 2-related factor 2; HO-1, heme oxygenase-1; ET-1, endothelin-1; mPTP, mitochondrial permeability transition pore; PAR 1, protease-activated receptor-1; CECs, cerebral endothelial cells; GLT-l, glutamate transporter-1; ERK, extracellular signal-regulated kinase.
